# Stimulation of *Acanthamoeba castellanii* excystment by enzyme treatment and consequences on trophozoite growth

**DOI:** 10.3389/fcell.2022.982897

**Published:** 2022-09-12

**Authors:** Zineb Fechtali-Moute, Philippe M. Loiseau, Sébastien Pomel

**Affiliations:** Université Paris-Saclay, CNRS, BioCIS, Orsay, France

**Keywords:** Acanthamoeba, free-living amoeba, excystment, cellulase, protease, trophozoite growth

## Abstract

*Acanthamoeba castellanii* is a widespread Free-Living Amoeba (FLA) that can cause severe ocular or cerebral infections in immunocompetent and immunocompromised patients, respectively, besides its capacity to transport diverse pathogens. During their life cycle, FLA can alternate between a vegetative form, called a trophozoite, and a latent and resistant form, called a cyst. This resistant form is characterized by the presence of a cell wall containing two layers, namely the ectocyst and the endocyst, mainly composed of cellulose and proteins. In the present work, we aimed to stimulate *Acanthamoeba castellanii* excystment by treating their cysts with a cellulolytic enzyme, i.e., cellulase, or two proteolytic enzymes, i.e., collagenase and pepsin. While 11 days were necessary to obtain total excystment in the control at 27°C, only 48 h were sufficient at the same temperature to obtain 100% trophozoites in the presence of 25 U/mL cellulase, 50 U/mL collagenase or 100 U/mL pepsin. Additionally, more than 96% amoebae have excysted after only 24 h with 7.5 U/mL cellulase at 30°C. Nevertheless, no effect of the three enzymes was observed on the excystment of *Balamuthia mandrillaris* and *Vermamoeba vermiformis*. Surprisingly, *A. castellanii* trophozoites excysted in the presence of cellulase displayed a markedly shorter doubling time at 7 h, in comparison to the control at 23 h. Likewise, trophozoites doubled their population in 9 h when both cellulose and cellulase were added to the medium, indicating that *Acanthamoeba* cyst wall degradation products promote their trophozoite proliferation. The analysis of cysts in epifluorescent microscopy using FITC-lectins and in electron microscopy revealed a disorganized endocyst and a reduction of the intercystic space area after cellulase treatment, implying that these cellular events are preliminary to trophozoite release during excystment. Further studies would be necessary to determine the signaling pathways involved during this amoebal differentiation process to identify new therapeutic targets for the development of anti-acanthamoebal drugs.

## Introduction

Free-living amoebae (FLA) are unicellular eukaryotes widely distributed in natural or artificial environments such as soil, rivers, swimming pools, or drinking water reservoirs (Rodriguez-Zaragoza, 1994; Thomas and Ashbolt, 2011). These protozoa have the ability to live and multiply autonomously in nature, in opposition to parasitic amoebae, such as *Entamoeba histolytica* requiring a host to survive (Martinez and Visvesvara, 1997). They constitute a polyphyletic group including Amoebozoa, the most diverse and the only one exclusively composed of amoebae, Rhizaria, Discoba, Opisthokonta, and others (Burki et al., 2020; Shi et al., 2021). Among them, FLA belonging to the genera *Acanthamoeba*, *Balamuthia*, *Naegleria*, *Sappinia* and *Vermamoeba* (previously named *Hartmannella*) can become pathogenic to human and cause severe ocular or cerebral infections (Aitken et al., 1996; Centeno et al., 1996; Visvesvara et al., 2007). Especially, *Acanthamoeba sp.* is able to provoke amoebic keratitis which can be treated by a combination of chlorhexidine with polyhexamethylene biguanide or propamidine but leads to a treatment failure in around 40% of the cases (Randag et al., 2019; Scruggs et al., 2022). Importantly, this FLA can also give rise to granulomatous amoebic encephalitis for which no effective monotherapy is presently available. Despite the use of current therapies empirically associating a large cocktail of drugs, a high mortality is observed, above 90% of the cases (Visvesvara et al., 2007; Taravaud et al., 2021).

During its life cycle, *Acanthamoeba sp.* can alternate between two life stages: the trophozoite, a vegetative form able to reproduce, feed and move via cytoplasmic extensions called pseudopods, and the cyst, a dormant and resistant form allowing the amoeba to survive in hostile environmental conditions (Khan, 2006). The formation of mature cyst requires three main steps: 1) rounding and immobilization of the trophozoite form, 2) dehydration during which the cell loses most of its fluids and decreases in volume, and 3) the formation of the double-layered cyst wall with the arrangement of the outer layer, the ectocyst, before the fibrillar inner layer, the endocyst, providing cell protection (Bowers and Korn, 1969). Encystment is regulated by several signaling pathways such as cAMP synthesis, MAPK pathway, autophagy, cellulose synthesis, or DNA methylation involving diverse enzymes or proteins such as kinases, proteases, methyltransferases, acetyltransferases, deacetylases or heat shock proteins (Moon et al., 2007; Moon et al., 2009; Moon et al., 2011a; Moon et al., 2011b; Moon et al., 2012a; Moon et al., 2012b; Fouque et al., 2012; Moon and Kong, 2012; Song et al., 2012; Moon et al., 2013; Moon et al., 2016; Moon et al., 2017; Joo et al., 2020; Rolland et al., 2020; Wang et al., 2021). More recently, a time-resolved multi-omics analysis has revealed a rapid phospho-regulation of cytoskeleton and translation factors during encystment (Bernard et al., 2022). The cyst wall of *A. castellanii* is mainly composed of acid-resistant proteins and cellulose, this latter component being present in both the endocyst and the ectocyst, especially in the inner layer where it represents one third of the dry weight (Weisman, 1976; Chavez-Munguia et al., 2005; Dudley et al., 2009). Conversely, *Acanthamoeba sp.* excystment starts with a cytoplasmic bud protruding through an ostiole, corresponding to a cyst pore where both endo- and ectocyst layers are in contact, allowing emergence of the trophozoite from the empty cyst wall (Chambers and Thompson, 1972; Chavez-Munguia et al., 2005). Although the pathways involved in *Acanthamoeba sp.* excystment are currently not well known, this cellular process is typically accompanied by the secretion of depolymerizing enzymes such as cellulase or proteases (Krishna-Murti and Shukla, 1984). Some studies have reported a cysticidal effect of cellulase and a subtilisin-like serine protease treatment on *Acanthamoeba sp.* and *Vermamoeba vermiformis*, respectively (Fouque et al., 2015a; Ortilles et al., 2017; Lazuana et al., 2019). Moreover, in several *Acanthamoeba* species, treatments by cellulase and pancreatic protease can hydrolyze more than half of cyst wall carbohydrates and proteins, respectively (Barrett and Alexander, 1977). However, to the best of our knowledge, no study in the literature has described an influence of cellulase or protease treatment on FLA excystment.

The *in vitro* culture of FLA isolated from the environment requires media with a wide range of different components depending on the FLA genera (Schuster, 2002), giving a limitative diversity of amoebae since only environmental FLA that are able to grow in these media will be selected (Denet et al., 2017). Moreover, even after using a panel of different media, some environmental FLA, isolated in the cyst form, were not able to develop in culture *in vitro*, indicating that they are either dead, or viable but non-culturable (Taravaud et al., 2018). In order to facilitate the *in vitro* development of FLA cysts isolated from the environment and thus to determine their viability, we attempted in the present work to gently digest their cyst wall in order to stimulate trophozoite release from cysts, and therefore their proliferation in culture. The effect of different conditions of treatment by cellulolytic and proteolytic enzymes was thus analyzed on FLA excystment. The influence of cellulase was further investigated on *Acanthamoeba castellanii* trophozoite growth following their release from cysts. Cellulase activity was then studied on *A. castellanii* cysts at the cellular and ultrastructural levels in epifluorescence microscopy using lectin labeling and in transmission electron microscopy, respectively.

## Materials and methods

### Chemicals

All chemicals were purchased from Merck Laboratories (St-Quentin-Fallavier - France), unless otherwise stated. The enzymes used in this study were cellulase from *Aspergillus niger*, chitinase from *Streptomyces griseus*, collagenase type I from *Clostridium histolyticum* and pepsin from porcine gastric mucosa and were purchased from Merck Laboratories (St-Quentin-Fallavier - France). Fetal Bovine Serum (FBS) was provided by Fisher Scientific (Illkirch, France).

### FLA culture

*Acanthamoeba castellanii* (ATCC 30010 strain) was grown axenically in PYG medium (ATCC medium 712) composed of 2% (w/v) peptone protein, 0.1% (w/v) yeast extract, 100 mM glucose, 4 mM MgSO_4_, 400 µM CaCl_2_, 3.4 mM sodium citrate, 2.5 mM Na_2_HPO_4_, 2.5 mM KH_2_PO_4_, 50 µM Fe (NH_4_)_2_(SO_4_)_2_, pH 6.5 (Magistrado-Coxen et al., 2019).

*Vermamoeba vermiformis* (ATCC 50237 strain) was cultured axenically in PYNFH medium (ATCC medium 1,034) containing 1% (w/v) bacto-peptone, 1% (w/v) yeast extract, 0.1% (w/v) RNA type VI from torula yeast, 34 µM folic acid, 1.5 µM hemin, 3.5 mM Na_2_HPO_4_, 2.7 mM KH_2_PO_4_, 10% (v/v) FBS, pH 6.5 (Fouque et al., 2015a).

*Balamuthia mandrillaris* (ATCC 50209 strain) was cultured in RPMI-1640 medium (Merck, Saint-Quentin-Fallavier, France) supplemented with 10% FBS and penicillin at 100 U/mL and streptomycin at 100 μg/ml (Fisher Scientific, Illkirch, France) in the presence of a monolayer of Vero cells (ATCC, Manassas, VA, USA), as previously described (Schuster and Visvesvara, 1996).

*A. castellanii* and *V. vermiformis* were grown at 27°C in the dark without shaking and *B. mandrillaris* was cultivated at 37°C with 5% of CO_2_ in the dark without shaking. All FLA were sub-cultured with a starting density at 5 × 10^4^ amoebae/mL twice a week in these conditions.

### Encystment

The encystment method was the same for the three FLA used in this study, namely *Acanthamoeba castellanii*, *Vermamoeba vermiformis* and *Balamuthia mandrillaris*, and was adapted from the method previously developed by Fouque et al., 2012. Briefly, 15 mL of a culture of FLA at 10^6^ trophozoites/mL were centrifuged at room temperature at 3,000 *g* for 10 min, before resuspension and 3 washes in Neff buffer containing 0.1 M KCl, 8 mM MgSO_4_, 0.4 mM CaCl_2_, 1 mM NaHCO_3_, 20 mM Tris-HCl, pH 8.8 (Neff et al., 1964). Amoebae were then incubated in 15 mL of Neff buffer at 27°C for 5 days, and then further treated for 10 min at room temperature with 0.5% (w/v) SDS (Sodium Dodecyl Sulfate) to eliminate the remaining trophozoite and pseudocyst forms which are sensitive to SDS, unlike mature cysts. The selected mature cysts were then washed 3 times in PAS (Page’s Amoeba Saline) buffer (ATCC medium 1,323) containing 1 mM Na_2_HPO_4_, 1 mM KH_2_PO_4_, 16 µM MgSO_4_, 27 µM CaCl_2_, 2 mM NaCl, and then stored at 4°C in PAS buffer.

### Excysment

Excystment experiments were performed in the presence or absence (control) of cellulase, collagenase and/or pepsin, with a starting density of 5 × 10^4^ cysts/mL in 2 mL of FLA culture media, namely PYG for *A. castellanii*, PYNFH for *V. vermiformis* and complete RPMI-1640 for *B. mandrillaris* in a 24 well plate. The conditions of enzymatic treatments, in terms of enzyme concentrations, temperatures and incubation times, are described in the text. At the end of each treatment condition, the total number of cells, including cysts and trophozoites, was counted using a Malassez counting chamber (Fisher Scientific; France, Illkirch), then the culture was centrifuged at 3,000 *g* for 10 min at room temperature and treated with 0.5% (w/v) SDS for 10 min at room temperature to determine the density of mature cysts using a Nageotte counting chamber (Fisher Scientific; France, Illkirch). The number of trophozoites was then calculated by subtracting the number of mature cysts to the number of total cells. Cyst and trophozoite densities were further converted in percentages in comparison to total cells, corresponding to 100%, in order to determine their proportions in the total amoebal population.

### Trophozoite growth

*A. castellanii* trophozoite growth was determined at 27°C, with a starting density of 5 × 10^4^ ameobae/mL, in PYG medium supplemented with or without (control) 7.5 U/mL cellulase, 1 g/L, 2.5 g/L or 5 g/L cellulose, 50 mM, 100 mM or 150 mM glucose, 1 g/L, 2.5 g/L or 5 g/L cellulose with 7.5 U/mL cellulase, and 1 g/L, 2.5 g/L or 5 g/L cellulose with 7.5 U/mL cellulase pre-incubated 24 h at 27°C in cell-free PYG medium before adding trophozoites. The cell density was determined each day for a period of 13 days, to allow observation of stationary phase in trophozoite growth curve after excystment, by cell counting under light microscope using a Malassez chamber.

### Fluorescence microscopy

The lectin labelling protocol was adapted from the method previously described by Elloway et al., 2004. Briefly, after an incubation of *A. castellanii* cysts at a density of 5 × 10^4^ cysts/mL in the presence or absence (control) of cellulase at 7.5 U/mL at 27°C for 48h, amoebae were centrifuged at 3,000 *g* for 15 min at room temperature. The pellet was then washed three times in buffer A containing 10 mM HEPES pH 7.5, 100 μM CaCl_2_, 100 μM MnCl_2_ and 100 μM MgCl_2_, and further resuspended in 200 μL of buffer A. A volume of 100 μL of a FITC-lectin (Fluorescein-5-isothiocyanate-conjugated lectin) was further added to the amoeba suspension at a final concentration of 10 μg/ml. The FITC-lectins used in the present study were: Con A (Concanavalin A), GSL II (*Griffonia Simplicifolia* Lectin II), Jacalin, LCA (*Lens Culinaris* Agglutinin), LEL (*Lycopersicon Esculentum* Lectin), SBA (SoyBean Agglutinin), VVL (*Vicia Villosa* Lectin, also called VVA), WGA (Wheat Germ Agglutinin) or SWGA (Succinylated Wheat Germ Agglutinin; Eurobio, Les Ulis, France). After 30 min of labeling at 4°C in the dark, the amoebae were centrifuged at 3,000 *g* for 10 min at 4°C, washed 3 times and further resuspended in buffer A. The FITC-lectin-labelled cysts were then observed with an inverted epifluorescence microscope Leica Dmi8 (Leica, Nanterre, France) equipped with a Leica DFC9000 GT cooled monochrome camera (4.2 megapixels) and controlled by the Leica Application Suite software (LAS X 3.7.0.20979). Images were further processed in ImagesJ (version 1.8.0_172, Bethesda, MD, USA).

### Transmission electron microscopy

The protocol of sample preparation for electron microscopy was adapted from Taravaud et al., 2017. After a treatment of *A. castellanii* cysts at a density of 5 × 10^4^ cysts/mL in the presence or absence (control) of cellulase at 5 U/mL at 27°C for 48h, amoebae were washed 3 times with PAS buffer, centrifuged at 3,000 *g* for 15 min at room temperature and fixed 2 h at room temperature with fixative buffer containing 3% (v/v) glutaraldehyde, 1% (v/v) para-formaldehyde, 0.1 M cacodylate pH 7.5. Cells were washed 3 times with 0.1 M cacodylate buffer pH 7.5, then incubated for 1 h at room temperature in 1% (w/v) osmium tetroxide pH 7.5 and further rinsed 3 times in 0.1 M cacodylate pH 7.5. The first inclusion was performed by putting pellets in 2% Low Melting Point (LMP) agarose (Merck, Saint-Quentin-Fallavier, France) to obtain concentrated pellets in 1 mm^3^ cubes. Then the pellets were dehydrated in graded acetone series: 50% (2 × 1 min), 70% (2 × 5 min), 90% (2 × 15 min) and 100% (4 × 15 min). Embedding in epoxy resin (Low Viscosity Premix Kit Medium, Agar Scientific, Oxford instruments, Oxford, United Kingdom) was performed in graded series (50–100%) in 2 days. Blocs were polymerized for 24 h at 60°C. Ultrathin sections (80 nm) were cut with an ultramicrotome EM UC6 (Leica Microsystems, Nanterre, France), collected on formvar carbon-coated copper grids, and then stained for 15 min with 2% uranyl acetate (Merck, Saint-Quentin-Fallavier, France), washed 3 times with water and treated with 3% Reynolds lead citrate (Reynolds, 1963) for 7 min before observation. Grids were washed 3 times with water before examination under a JEM 1400 TEM operating at 120 kV (JEOL). TEM Images were acquired using a post-column high-resolution (9 megapixels) high-speed camera (RIO9; Gatan, AMATEK, Elancourt, France) and processed with Digital Micrograph software (GMS3, Gatan, AMATEK, Elancourt, France). Vesicle and intercystic space areas were measured by using the option “Polygon selections” in ImageJ software (version 1.8.0_172; USA, Bethesda, MD).

### Statistical analyses

Non-parametric two-tailed Mann-Whitney test (*p* < 0.05) was performed for comparisons between groups. All statistical analyses were performed with GraphPad Prism (version 9.3.1; San Diego, CA, USA).

## Results

**FIGURE 1:**
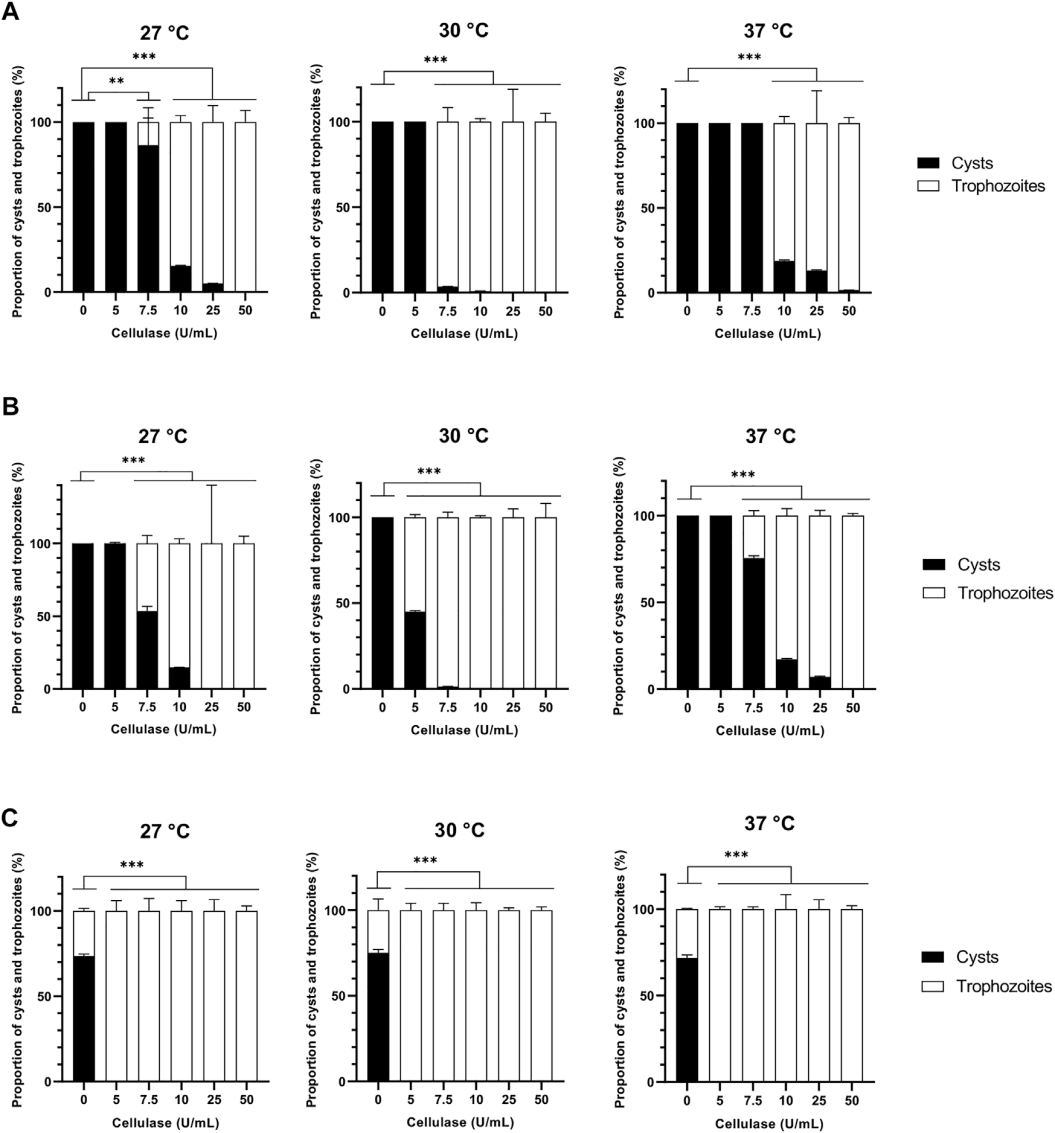
Effect of cellulase on *Acanthamoeba castellanii* excystment. Proportions of *A. castellanii* trophozoites and cysts observed following 24 h **(A)**, 48 h **(B)** and 72 h **(C)** of cellulase treatment at 27, 30 and 37°C as a function of enzyme concentration. The results correspond to the mean of four independent experiments ±SD.

The purpose of this work was to analyze the effect of a slight digestion of *A. castellanii* cysts by cellulase and proteases on their excystment, cellulose and proteins being the main components of *Acanthamoeba sp.* cyst walls (Weisman, 1976). As a cysticidal effect was previously observed with cellulase at concentrations of 500 U/mL and 1500 U/mL on *Acanthamoeba sp.* (Ortilles et al., 2017; Lazuana et al., 2019), we treated *A. castellanii* cysts with a range of lower concentrations, from 5 U/mL to 50 U/mL, in order to avoid cyst death. Different conditions of temperatures, from 27 to 37°C, and incubation times, from 24 to 72 h, were also evaluated in order to determine optimal conditions of treatment (Figure 1). At 24 h of treatment, at all temperatures analyzed, no excystment of *A. castellanii* was observed at 5 U/mL, as for the untreated control (Figure 1A). However, at the same incubation time with 7.5 U/mL cellulase, more than 96% of the amoebae were in the trophozoite form at 30°C, while no or very little effect was observed at the other temperatures (Figure 1A). Moreover, from 10 U/mL to 50 U/mL of cellulase more than 95% of the amoebae were in the trophozoite form at 24 h of treatment at all temperatures evaluated (Figure 1A). At 48 h of treatment, 100% of the amoebae were in the cyst form in the untreated control at 30°C, while 55% and more than 98% of the amoebae were in the trophozoite form at the lowest cellulase concentration of 5 U/mL and in the range from 7.5 U/mL to 50 U/mL, respectively (Figure 1B). However, at the same incubation time, at 27°C and 37°C, excystment started only at 7.5 U/mL, with 24–46% of trophozoite forms observed, and became considerable, with more than 82% of trophozoites, at higher concentrations (Figure 1B). Interestingly, no effect was observed on *A. castellanii* excystment with treatments in the same conditions of temperatures and incubation times for chitinase, a glycoside hydrolase acting on chitin, a polymer of N-acetyl-β-D-glucosamine distinct from the chains of D-glucose constituting cellulose (Supplementary Figure S1; Linder et al., 2002; Derda et al., 2009).

**FIGURE 2:**
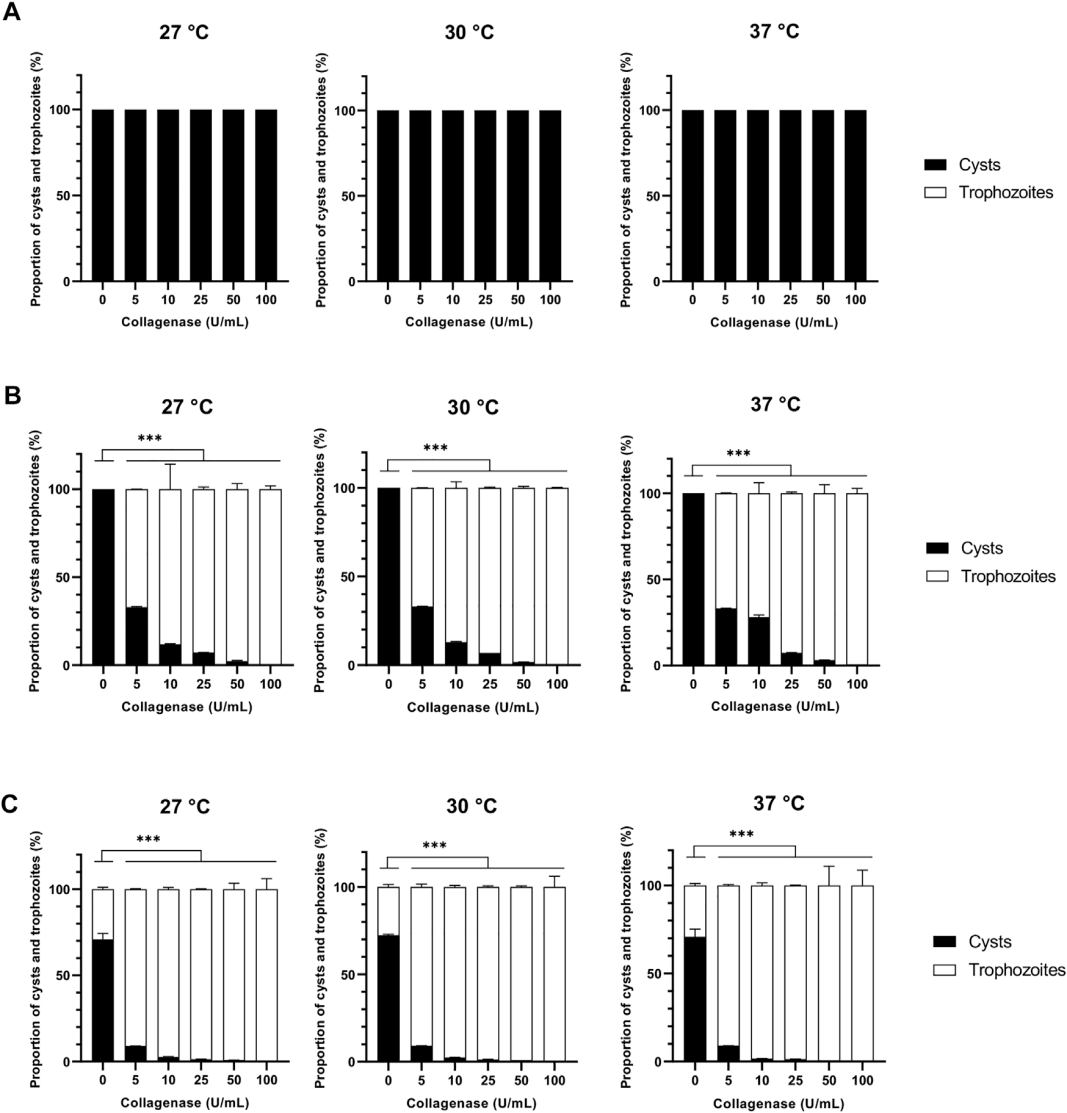
Effect of collagenase on *Acanthamoeba castellanii* excystment. Proportions of *A. castellanii* trophozoites and cysts observed following 24 h **(A)**, 48 h **(B)** and 72 h **(C)** of collagenase treatment at 27, 30 and 37°C as a function of enzyme concentration. The results correspond to the mean of four independent experiments ±SD.

**FIGURE 3:**
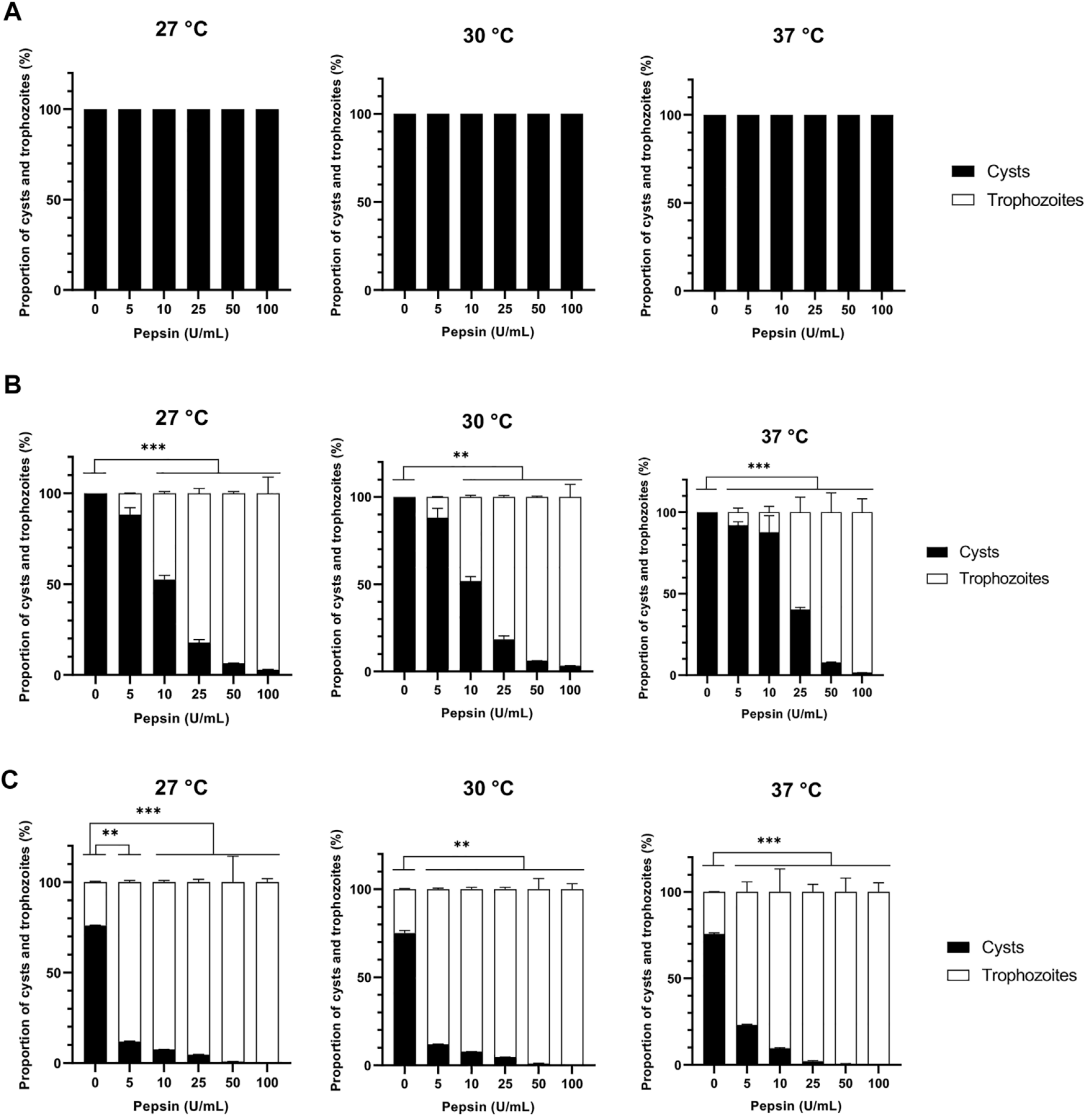
Effect of pepsin on *Acanthamoeba castellanii* excystment. Proportions of *A. castellanii* trophozoites and cysts observed following 24 h **(A)**, 48 h **(B)** and 72 h **(C)** of pepsin treatment at 27, 30 and 37°C as a function of enzyme concentration. The results correspond to the mean of four independent experiments ±SD.

The proteolytic enzymes selected in this work were two proteases from distinct classes, namely collagenase and pepsin belonging to metalloproteases and aspartic proteases, respectively (Elsasser and Goettig, 2021). No effect was observed for both proteases on *A. castellanii* excystment at 24 h of treatment at all temperatures and concentrations used (Figure 1A, Figure 2A). After 48 h of collagenase treatment, at 27, 30 and 37°C, excystment was noticed from the lowest concentration of 5 U/mL with a proportion of 67% of trophozoites, which increased at 72–88% at 10 U/mL, and was above 90% from 25 U/mL to 100 U/mL (Figure 2B). After 48 h of pepsin treatment, at all temperatures used, 88%–92% of cysts were observed at 5 U/mL, and 60%–82% of trophozoites from the concentration of 25 U/mL (Figure 3B). Nonetheless, at the same treatment time, at 10 U/mL the proportion of excystment was different depending on the temperature: around half of the amoebae were already in the trophozoite form at 27°C and 30°C, while only 12% of this amoeba form were observed at 37°C (Figure 3B).

After 72 h of treatment, at 27°C, 30°C and 37°C, and all concentrations used for the 3 enzymes, more than 76%, above 90% and 100% of the pepsin-, collagenase- and cellulase-treated amoebae were in the trophozoite form, respectively, while only 27–36% of trophozoites was observed in the untreated control (Figure 1C, Figure 2C, Figure 3C). In the absence of treatment at 27°C, a total excystment, with 100% trophozoites and no cysts, was only observed after 11 days of incubation in culture medium (Figure 6B). These results show that the 3 enzymes used in the present study can accelerate considerably the cellular process of amoeba excystment, with the following optimal conditions to obtain more than 96% excysted trophozoites in the shortest period of time with the lowest enzyme concentration: 7.5 U/mL for 24 h at 30°C for cellulase, 50 U/mL for 48 h at 27°C, 30°C or 37°C for collagenase, and 100 U/mL for 48 h, also independently of the temperature, for pepsin (Table 1).

**TABLE 1:** Summary of the optimal conditions of enzyme treatment for *A. castellanii* excystment. Optimal condition is defined as the enzyme treatment condition where more than 96% amoebae have excysted in the shortest period of time with the lowest enzyme concentration.

	Cellulase	Collagenase	Pepsin
Concentration	7.5 U/mL	50 U/mL	100 U/mL
Time	24 h	48 h	48 h
Temperature	30°C	27–37°C	27–37°C

Remarkably, no trophozoite of *A. castellanii* was observed after incubation of cysts at 30°C after up to 96 h in the presence of 100 U/mL pepsin in a culture medium at pH 2, corresponding to the optimal pH of this enzyme. These cysts did not further excyst and develop as a trophozoite culture after a passage in a regular culture medium without pepsin, in opposition to pepsin-untreated control at pH 2 which excysted and presented 100% of the trophozoite form after 18 days, corresponding to 7 days later than the untreated control at pH 6.5 (Supplementary Figure S2).

**FIGURE 4:**
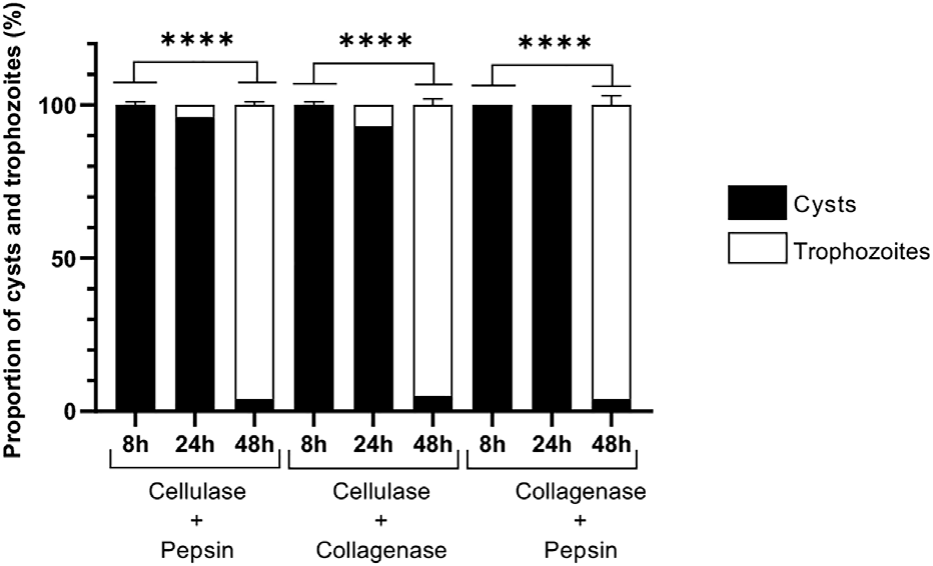
Effect of enzyme combinations on *Acanthamoeba castellanii* excystment. Proportions of *A. castellanii* trophozoites and cysts observed following 8, 24 and 48 h of treatment by enzyme associations at 30°C. In these associations, all enzyme concentrations were at 5 U/mL. The results correspond to the mean of three independent experiments ±SD.

In addition, treatments with two by two associations of cellulase, collagenase and pepsin were further investigated in order to determine any supplemental effect on excystment kinetics (Figure 4). Cysts were therefore treated with enzyme associations at 30°C with individual enzyme concentrations at 5 U/mL and were further analyzed after 8 h, 24 h, and 48 h (Figure 4), as no excystment was observed in these conditions for the three enzymes after 24 h of treatment (Figure 1A, Figure 2A, Figure 3A). For all enzyme associations at 30°C, 100% cysts was observed after 8 h, and above 95% of trophozoites after 48 h (Figure 4). However, only 12–67% of trophozoites was observed for individual enzymatic treatments at this latter time of incubation (Figure 1B, Figure 2B, Figure 3B). Moreover, after 24 h at 30°C, while 100% cysts was observed when both proteases were associated, trophozoites started to be observed, with less than 10%, when cellulase was combined with either collagenase or pepsin (Figure 4). These data indicate that all enzyme associations, and especially the combination of cellulase with either collagenase or pepsin, have an additive effect on *A. castellanii* excystment kinetics.

**FIGURE 5:**
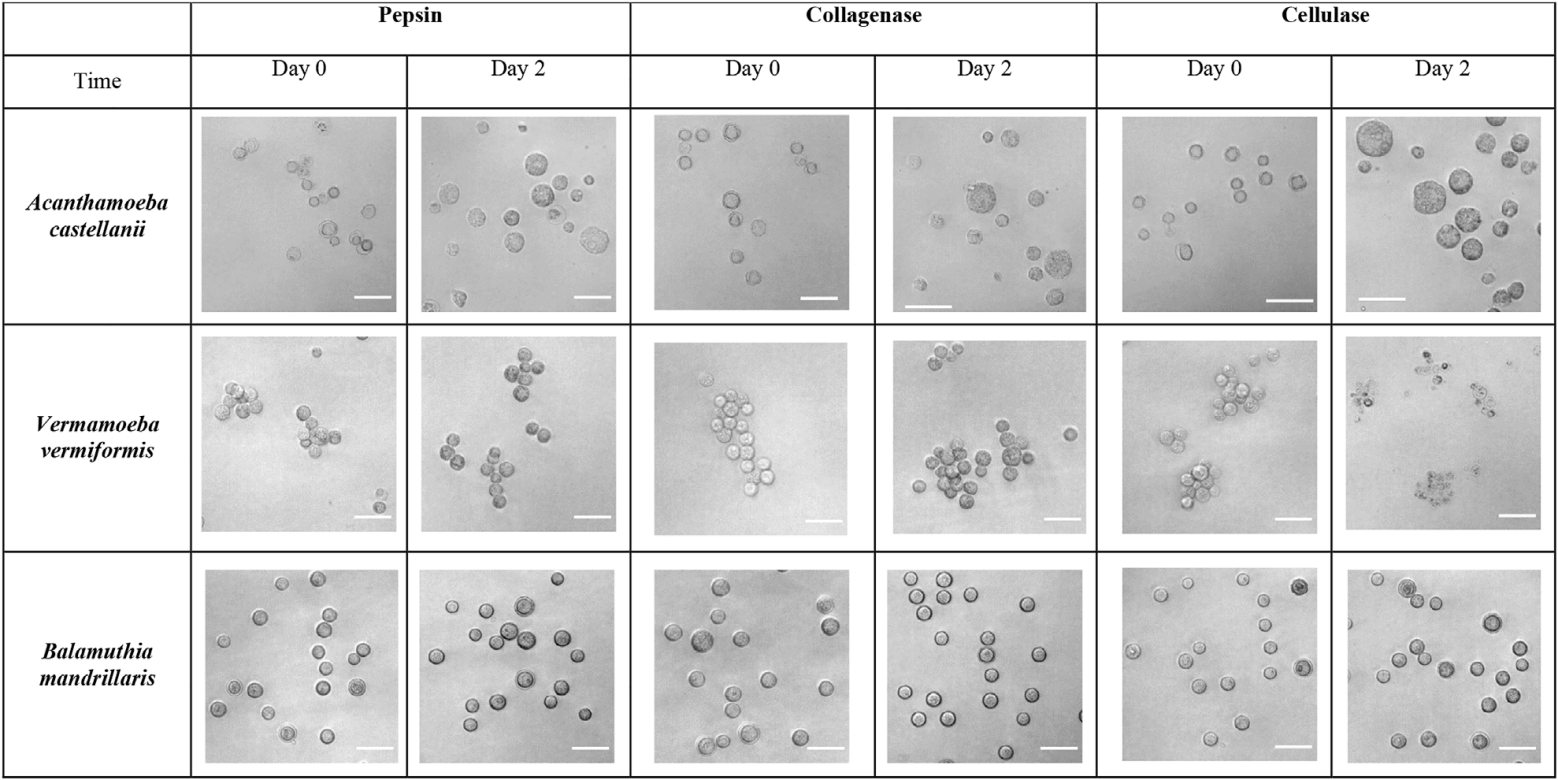
Effect of cellulase, collagenase and pepsin on the excystment of *Acanthamoeba castellanii*, *Vermamoeba vermiformis*, and *Balamuthia mandrillaris*. Phase contrast observation of *A. castellanii*, *V. vermiformis*, and *B. mandrillaris* before (Day 0) and after treatment of their cysts for 2 days at 30°C by 100 U/mL pepsin, 50 U/mL collagenase, or 7.5 U/mL cellulase. Scales bars = 20 µm.

The action of cellulase, collagenase and pepsin treatment was also investigated on the excystment of two other FLA, *Vermamoeba vermiformis* and *Balamuthia mandrillaris*, in the optimal conditions determined for *A. castellanii* (Figure 5). In these conditions, no effect was observed of all 3 enzymes on *B. mandrillaris* excystment, as only cysts were observed after 2 days of treatment (Figure 5). Likewise, on *V. vermiformis*, no trophozoite was observed with collagenase and pepsin (Figure 5). Moreover, these *B. mandrillaris* and *V. vermiformis* treated cysts were able to further excyst and grow in a culture medium, showing their viability (data not shown). However, *V. vermiformis* cysts incubated with cellulase were completely lysed after treatment (Figure 5).

**FIGURE 6:**
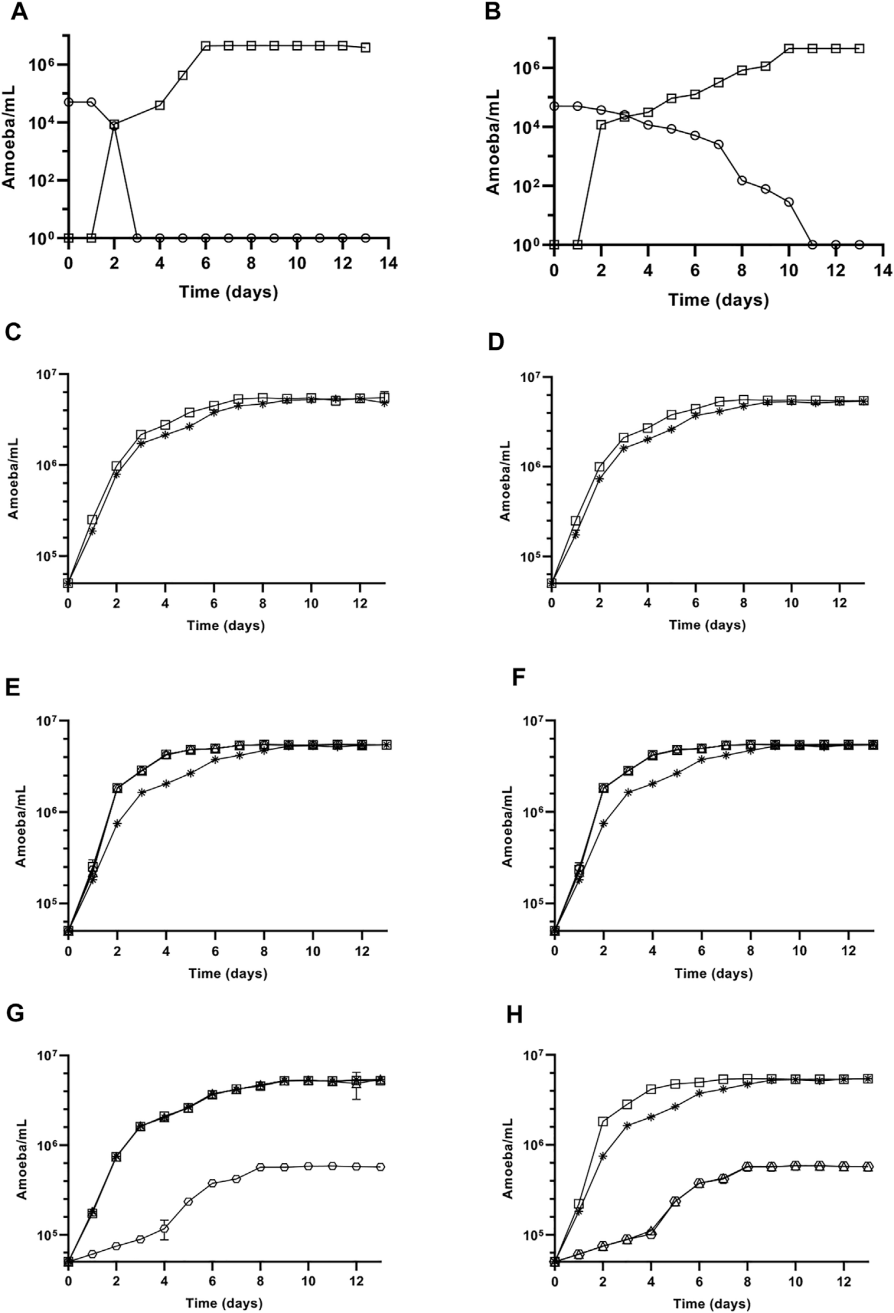
Influence of cellulase and cellulose on *A. castellanii* trophozoite growth at 27°C. **(A,B)** Cyst (○) excystment and trophozoite (□) growth kinetics in the presence **(A)** or absence **(B)** of 7.5 U/mL cellulase. **(C,D)** Growth kinetics of trophozoites in the presence (□) or absence (*) of 7.5 U/mL cellulase after a passage following their excystment in the presence **(C)** or absence **(D)** of 7.5 U/mL cellulase. **(E,F)** Growth kinetics of non-freshly excysted, regularly passed, trophozoites in PYG medium alone (*) or PYG medium supplemented with 7.5 U/mL cellulase and cellulose at 1 g/L (□), 2.5 g/L (△) or 5 g/L (○), either directly to the trophozoites **(E)**, or pre-incubated 24 h at 27°C before adding the amoebae **(F). (G,H)** Growth kinetics of regularly passed trophozoites in PYG culture medium alone (*) or PYG medium supplemented with cellulose **(G)** at 1 g/L (□), 2.5 g/L (△), or 5 g/L (○), or with glucose **(H)** at 50 mM (□), 100 mM (△), or 150 mM (○). The results correspond to the mean of three independent experiments ±SD.

Thereafter, the action of cellulase was investigated further as this enzyme showed a more rapid effect on *A. castellanii* excystment after 24 h of treatment, in comparison to both collagenase or pepsin (Figures 1–3). In particular, trophozoite growth was analyzed after excystment in the presence of 7.5 U/mL cellulase at 27°C, in comparison to the untreated control (Figures 6A,B). Intriguingly, while trophozoite stationary phase was obtained with a similar amoebal density at approximately 4.5 × 10^6^ amoebae/mL in both conditions, this phase was reached at the 6th day of treatment in the presence of cellulase, with a very rapid doubling time of 7 h, and only at the 10th day in the untreated control, with a doubling time of only 23 h (Figures 6A,B). Additionally, after a passage following excystment in the presence or absence of cellulase, no growth difference was observed with trophozoites cultured with or without cellulase (Figures 6C,D). However, when non-freshly excysted, regularly passed, trophozoites were grown in the presence of cellulose and cellulase either pre-incubated for 24 h in the culture medium at 27°C to allow cellulose digestion before adding the amoebae (Figure 6F), or directly supplemented to the cells without pre-incubation (Figure 6E), the doubling time was likewise shorten at 9 h in both conditions, in comparison to the untreated control where 14 h were necessary to double the cell population. This growth stimulation effect was observed at all cellulose concentrations used, from 1 g/L to 5 g/L, in the presence of 7.5 U/mL cellulase (Figures 6E,F). Interestingly, trophozoite doubling time was also shorten at 9 h when the culture medium was supplemented with 50 mM glucose, but their growth was negligible when additive glucose concentration was increased at 100 mM (Figure 6H). Furthermore, trophozoite growth was similar to the control in the presence of 1 g/L and 2.5 g/L cellulose alone, while a very little development was observed with 5 g/L cellulose (Figure 6G).

**FIGURE 7:**
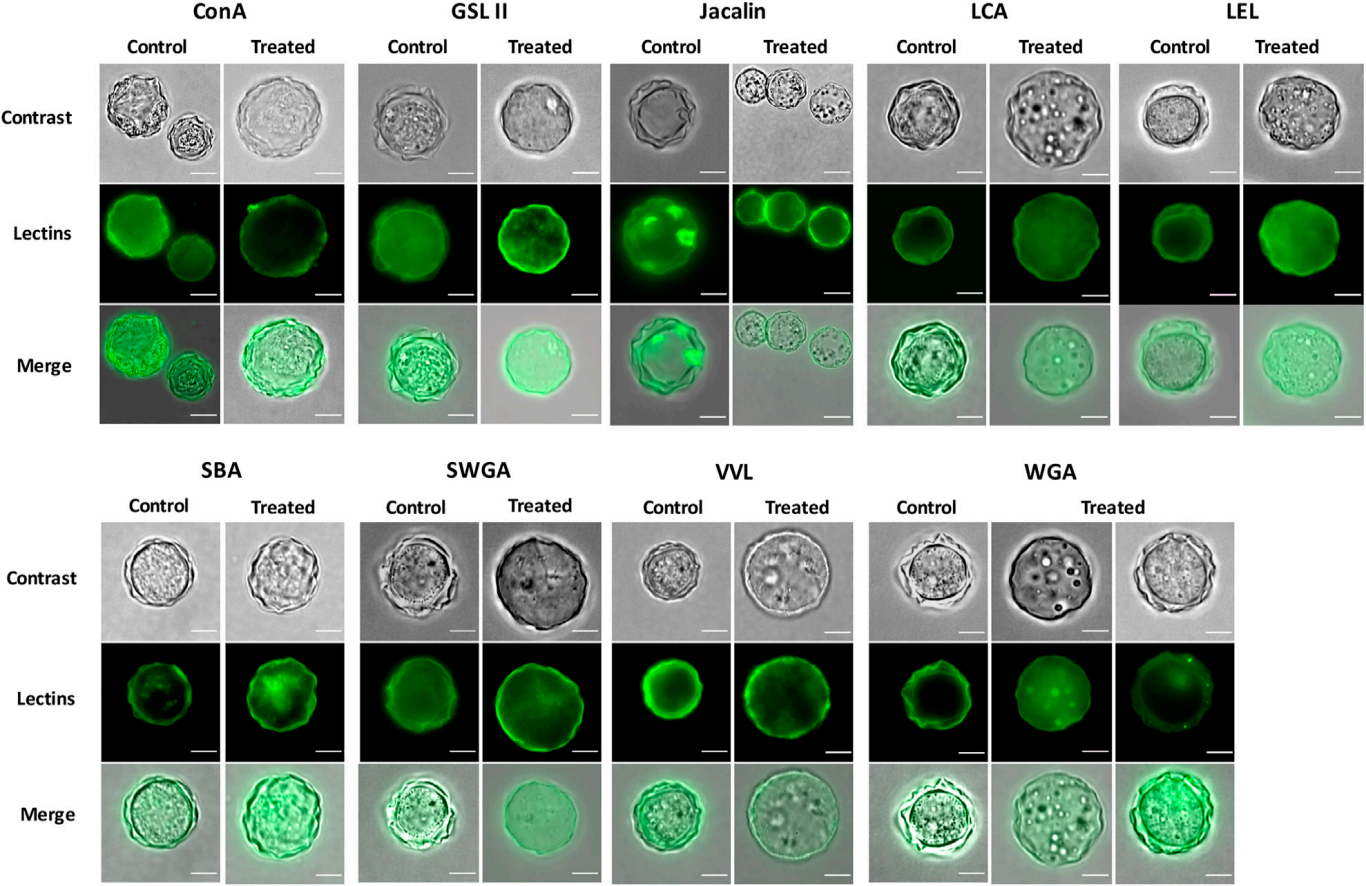
Influence of cellulase treatment on carbohydrate localization in *A. castellanii* cysts. After a treatment in the presence or absence (control) of 7.5 U/mL cellulase at 27°C for 48 h, carbohydrates were labeled by FITC-lectins (green). Phase contrast and merge images are shown on top and at the bottom of the FITC-lectin image, respectively. Nine lectins were used in this experiment: Con A, GSL II, Jacalin, LCA, LEL, SBA, SWGA, VVL and WGA. Scale bars = 10 µm.

In order to analyze the distribution of glycopolymers in *A. castellanii* cyst walls following cellulase treatment in comparison to untreated control, a FITC-coupled lectin labeling was performed following treatment for 48 h at 27°C with 7.5 U/mL cellulase, where approximately 50% of trophozoites was observed (Figure 7). Nine different FITC-lectins previously described to have affinities to distinct monosaccharides were used in this study (Elloway et al., 2004; Figure 7). Among the nine lectins used, no loss or decrease of fluorescent signal intensity was observed after cellulase treatment. Moreover, the ectocyst of cellulase-treated *A. castellanii* cysts was similarly labeled in comparison to untreated control (Figure 7). However, while the endocyst was labeled in half of the cysts at best in the control, a tendancy to a decrease in the proportion of endocyst-labeled cysts was observed in cellulase-treated amoebae with all lectins (Supplementary Figure S3; Figure 7). Additionally, intracellular vesicles were revealed after cellulase treatment with GSL II, LCA and WGA and were labeled before and after treatment with SBA (Figure 7). Ostioles were also clearly labeled by Jacalin in untreated control, while they were not, or only partially, recognized by this lectin after treatment (Figure 7). In our conditions, trophozoites were not labeled by any of the lectin used, except for SWGA which revealed intracellular vesicles in this amoeba stage (Supplementary Figure S4). Furthermore, small dots were revealed in the intercystic space with WGA after cellulase treatment (Figure 7).

**FIGURE 8:**
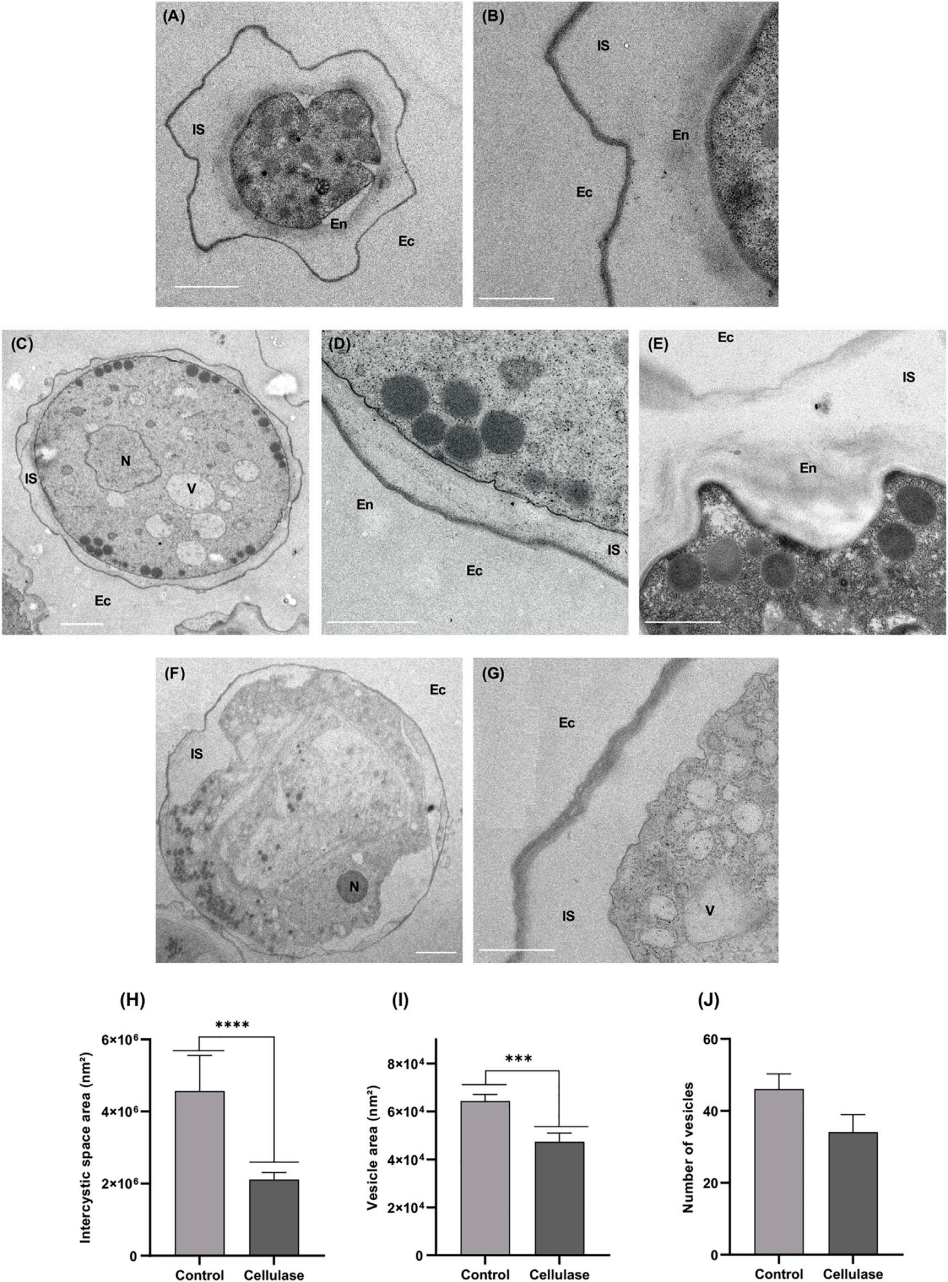
Analysis in transmission electron microscopy of *A. castellanii* cysts treated at 30°C for 48 h in the absence **(A,B)** or in the presence **(C–G)** of 5 U/mL cellulase. Treated cysts are characterized by a reduction of the intercystic space **(C,D,F,G)** in comparison to the control **(A,B)**, with an endocyst either scarcely visualizable **(C,D)** or absent **(F,G)**. Some treated cysts displayed a distance between endocyst and ectocyst comparable to the control, but with a disturbed endocyst and the presence of fibrillar material released within the intercystic space **(E)**. In treated cysts, lipidic vesicles were mostly localized at the periphery of the amoeba **(C,F)**, in opposition to the control **(A)**. En, Endocyst; Ec, ectocyst; IS, Intercystic Space, N, Nucleus, V, Vacuole; White arrow: lipidic vesicle; Double black arrow: fibrillar material within the intercystic space. Scale bars represent 2 µm **(A,F)**, and 1 µm **(B,C,D,E,G)**. The intercystic space area **(H)** as well as the lipidic vesicle area **(I)** and number **(J)** were determined in cellulase-treated and untreated amoebae. The results correspond to the mean of 30 cells ±SEM. Three independent experiments were performed.

At the ultrastructural level, the area of the space between the ectocyst and the endocyst, called the intercystic space, was significantly reduced in most of the cellulase-treated cysts, and was estimated to be twice smaller compared to untreated control (Figures 8A–D, Figures 8F–H). Moreover, the endocyst was either hardly visible (Figures 8C,D) or absent (Figures 8F,G) in the cellulase-treated cysts where the intercystic space area was reduced. Nevertheless, some cellulase-treated cysts presented a loose disorganized endocyst with the presence of fibrillar material within an intercystic space comparable in size to the one of the control (Figure 8E). Additionally, cellulase-treated cysts displayed electron-dense vesicles, probably lipidic vesicles, mostly at the periphery of the amoeba while they were distributed within the cytoplasm of untreated cells (Figures 8A,C,F). These vesicles were significantly smaller in cellulase-treated cysts in comparison to the control, with a reduction of approximately 25% of their area (Figure 8I). However, the number of lipidic vesicles per amoeba did not vary significantly between treated and untreated cysts (Figure 8J).

## Discussion

As *Acanthamoeba sp.* cyst walls are mainly constituted of cellulose and proteins, we attempted in the present work to digest these components with cellulolytic or proteolytic enzymes with the aim to promote trophozoite excystment. Based on their sequence similarities, structural and catalytic properties, cellulases can be found in 11 Glycoside Hydrolase (GH) families while having similar substrate specificities (Lakhundi et al., 2015). Although the cellulase from *Aspergillus niger* used in the current study belongs to the glycoside hydrolase family 12, the cellulases of *Acanthamoeba sp.* have been identified to belong to glycoside hydrolase family 5 (Lakhundi et al., 2015). However, both glycoside hydrolase families 5 and 12 have been described to have similar structural and catalytic activities (Segato et al., 2014), showing that the cellulase of *A. niger* could mimic the activity of the counterpart encoded in *A. castellanii*. Moreover, based on their catalytic mechanism, proteases have been classified in 5 major different classes: cysteine, serine, threonine, aspartic and metallo-proteases (Lopez-Otin and Bond, 2008). While the two last classes use an activated water molecule as a nucleophile to attack protein peptide bond, in the other enzymes, the nucleophile is an amino acid directly located within the active site of the proteases, leading to a higher reactivity order and more efficient mechanism (Cuesta et al., 2020; Elsasser and Goettig, 2021). In *Acanthamoeba sp.*, a subtilisin-like serine protease has been previously described to have a role in the excystment process (Dudley et al., 2008; Fouque et al., 2012). However, a treatment of *V. vermiformis* cysts by subtilisin-like protease with a concentration as low as 0.625 U/mL has been previously described to lead to high cysticidal activity (Fouque et al., 2015a). In order to avoid a cysticidal effect with protease treatment, we used in the current study lower efficient proteases belonging to the metallo- and aspartic protease class, namely collagenase and pepsin, respectively. Moreover, as both serine proteases and collagenases have been described to have a role in *Acanthamoeba sp.* pathogenicity (De Sousa-Carvalho et al., 2011), it was of interest to analyze also the influence of collagenase on *A. castellanii* excystment. In addition, pepsin was used in non-optimal conditions, at pH 6.5, which is distant from its optimal pH at 2 and where only a residual activity has been previously observed (Salelles et al., 2021), thus favoring a gentle digestion of the FLA cyst wall. Indeed, at pH 2, no excystment was observed in our conditions after an incubation of up to 18 days at 30°C in the presence of 100 U/mL pepsin, while the amoebae were able to excyst after a treatment in the same conditions at pH 2 without the enzyme, showing a cysticidal effect of pepsin at pH 2 on *A. castellanii*.

In order to avoid any toxic effect of enzyme treatment, cellulase was used with a range from 5 U/mL to 50 U/mL, much lower than the concentrations of 250 U/mL and 1500 U/mL previously described to cause less than 20% and less than 50% of cyst death, respectively (Lazuana et al., 2019), and proteases were used with a similar range, from 5 U/mL to 100 U/mL. As a result, in comparison to untreated *A. castellanii* cysts which need 11 days to completely excyst at 27°C, only 48 h were sufficient to give more than 96% of excystment at the same temperature in the presence of 50 U/mL of collagenase, 100 U/mL pepsin or 25 U/mL cellulase. These results show that, in these treatment conditions, cellulase, pepsin and collagenase can promote *A. castellanii* trophozoite release from cysts, with an acceleration of the excystment process of 8 days, corresponding to 73% of the excystment time, in comparison to untreated cysts. Moreover, at 30°C with 7.5 U/mL cellulase, a quasi-total excystment can be obtained after only 24 h, showing a temperature-dependent activity for this enzyme, which is less marked for collagenase and pepsin. The faster effect with smaller concentration of cellulase could be due to a higher specific activity of this enzyme in comparison to collagenase and pepsin in our conditions, and also to the high amount of cellulose present in *Acanthamoeba sp.* cyst wall (Weisman, 1976; Dudley et al., 2009). Moreover, the additive effect of the two by two associations of cellulase, collagenase and pepsin could be assigned to a complementary action of the enzymes on carbohydrates and proteins within the amoeba cyst wall leading to a faster excystment in comparison to cysts treated with individual enzymes: almost all amoebae have excysted after 48 h at 30°C with only 5 U/mL of each enzyme.

Remarkably, in the optimal conditions identified for *A. castellanii*, no effect of cellulase, collagenase or pepsin was observed on *V. vermiformis* or *B. mandrillaris*. This difference of activity could be ascribed to a distinct cell wall composition in these FLA. Indeed, the cyst wall of *Hartmanella glebae*, a FLA closely related to *V. vermiformis*, has been described to be composed of 64.7% proteins, and only 4.2% cellulose (Upadhyay et al., 1984). Moreover, the cyst wall of *B. mandrillaris* is presumably composed of a mixture of proteins and cellulose, but with a proportion that remains to be determined (Siddiqui et al., 2009a), although its carbohydrate composition has been identified with 20.9 mol% mannose and 79.1 mol% glucose (Siddiqui et al., 2009b). Because of differences of composition, the action of the enzymes could be prevented due to hindered sites of digestion, leading to an absence of activity on excystment. Conversely, some substrates could be easier to reach leading to an exacerbation of the enzyme effect, and thus to cyst lysis, as it was the case with cellulase on *V. vermiformis* in our study, indicating that cellulose has a crucial role in this FLA cyst wall integrity, even if it is present in minor proportion in this amoebal structure (Upadhyay et al., 1984).

Surprisingly, following their release from cysts, trophozoite growth was greatly stimulated in the presence of cellulase with a doubling time of 7 h, in comparison to untreated amoebae which doubled their population only every 23 h. However, no growth difference was observed on trophozoite growth after a passage in the presence or absence of cellulase following amoebae excystment, showing that cellulase did not have a direct effect on *A. castellanii* trophozoite proliferation. A hypothesis would be that cellulase added in the medium would help amoebae to digest their cyst wall to use it as a source of energy and stimulate trophozoite growth. When cellulase and cellulose were both added on trophozoites, either pre-incubated 24 h at 27°C prior to amoebae addition or directly added to the culture, doubling time was also markedly shortened to 9 h in comparison to untreated control, showing that the degradation product of cellulose can stimulate *A. castellanii* growth. Likewise, the addition of 50 mM glucose, the final product of cellulose degradation, to the culture medium stimulated *A. castellanii* growth with a doubling time of 9 h while no effect was observed with cellulose alone at 1 g/L and 2.5 g/L, and an inhibition of proliferation was obtained with higher concentrations of glucose, at 100 mM (equivalent to 18 g/L) and 250 mM (equivalent to 45 g/L), and cellulose at 5 g/L, showing a toxicity of these carbohydrates at these concentrations in the culture medium. Altogether, these results show that while cellulose or cellulase alone does not have any effect on amoeba growth, the product of cellulose degradation by cellulase promotes their proliferation. Therefore, following their excystment in the presence of cellulase, trophozoite growth is promoted by the degradation product of cellulose, mainly present in amoeba cyst wall, showing that the major carbohydrate resulting from cyst wall degradation after cellulase treatment, essentially glucose, can be used, at up to a supplemental concentration of 50 mM in the culture medium, as an energy source by trophozoites to promote their proliferation after excystment. Moreover, as empty cyst walls are no longer visible in culture following *A. castellanii* excystment in the absence of cellulase, it is conceivable that trophozoites would secrete their own cellulase during this cellular process allowing cyst wall degradation and amoebae proliferation, but with a much lower efficiency than in the presence of an additional cellulase. These data are in agreement with the studies previously describing the release of cellulase as well as proteases during *Acanthamoeba culbertsoni* excystment (Lasman 1975; Kaushal and Shukla, 1978a; Kaushal and Shukla, 1978b; Krishna-Murti and Shukla, 1984; Kaushal and Shukla, 1976). Moreover, as the monosaccharide composition of *Acanthamoeba* cyst wall is mainly constituted of glucose and galactose (Anwar et al., 2019), these carbohydrates could be released after cyst wall digestion during excystment and further used as an energy source or to synthesize glycoconjugates within the trophozoite. Likewise, in *V. vermiformis*, trophozoites have been described to digest its own cyst wall during excystment (Fouque et al., 2015b). Moreover, the difference of doubling times between trophozoites just after their release from cysts, at 23 h, and non-freshly excysted trophozoites, at 14 h, could be due to a difference of activity of their metabolism after excystment in comparison to regularly passed amoebae. Furthermore, the shorter doubling time of 14 h obtained in the present study with *A. castellanii* trophozoites in comparison to the one previously reported of 22 h (Taravaud et al., 2017), may be explained by presence of sodium citrate in the culture medium in the current work, which is recommended by ATCC and is included in diverse studies regarding *Acanthamoeba sp.* culture (Moon et al., 2018; Nakagawa et al., 2019; Li et al., 2020; Weber-Lima et al., 2020).

No effect was observed on *A. castellanii* excystment with chitinase in our study. However, only small chitinase concentrations, ranging from 0.1 to 2 U/mL, were possible to be used in our conditions, indicating a potential absence of significant activity in our study. The presence of chitin in *Acanthamoeba* cyst wall is currently controversial (Linder et al., 2002; Derda et al., 2009; Magistrado-Coxen et al., 2019). Nevertheless, several lectins used in the present study, i.e., WGA, SWGA and LEL, displayed a labeling of both ectocyst and endocyst layers in untreated cysts while they were previously described to have a better affinity for N-acetyl-β-D-glucosamine, the main component of chitin (Elloway et al., 2004). These results could be assigned to the ability of these lectins to bind to other carbohydrates in *Acanthamoeba* cyst wall (Immel et al., 2016), or to the presence of chitin in the cyst wall.

The FITC-lectin labeling revealed a tendancy to a decrease of endocyst recognition in cellulase-treated cysts, in comparison to untreated amoebae. This result was confirmed in electron microscopy where a disorganization of the endocyst was observed in cellulase-treated cysts. Moreover, the presence of fibrillar material in electron microscopy as well as WGA-labeled structures in epifluorescence microscopy within the intercystic space confirmed the endocyst breakdown following cellulase treatment. The deposition of fibrillar material within the intercystic space has also been observed during *Acanthamoeba lugdunensis* encystment (Garajova et al., 2019), indicating that the endocyst arrangement and disarrangement would originate from and lead to the deposition of fibrillar material within the intercystic space, respectively. Moreover, during encystment, both cellulose synthase and xylose isomerase have been reported to be involved in cyst wall formation (Aqeel et al., 2013; Bernard et al., 2022). In our study, besides the endocyst breakdown, a significant reduction of the intercystic space area was observed in the cellulase-treated cysts, probably due to a re-hydration of the amoeba upon excystment. Indeed, during *A. castellanii* encystment, a dehydration of the amoeba was previously described leading to a very dense cytoplasm in the cyst and a reduction of the cell volume (Bowers and Korn, 1969). In opposition to the dogma postulating that cysts are metabolically inactive, a recent study has reported that phosphate transport is more important in *A. castellanii* cysts than in trophozoites, and would be presumably used for anaerobic ATP synthesis and thus for energy metabolism in these resistant amoeba forms (Carvalho-Kelly et al., 2022), suggesting that amoebae in their cyst form can actively sense their environment to determine when the excystment process can be initiated. As some cellulase-treated cysts appeared with a disorganized endocyst, but with an intercystic space area comparable to the untreated control, it is probable that the endocyst would be disrupted prior to amoeba re-hydration and intercystic-space reduction during excystment. Following these cellular processes, the amoeba would emerge by a cytoplasmic bud through an ostiole in order to complete its excystment, as previously described (Chambers and Thompson, 1972; Chavez-Munguia et al., 2005). This *A. castellanii* excystment process is however different from *V. vermiformis* where no ostiole has been described in the cyst wall and which presumably relies on the digestion on its own cyst wall for trophozoite release (Fouque et al., 2015b).

Furthermore, some vesicules were revealed by GSLII, LCA and WGA labelings after cellulase treatment in epifluorescence microscopy, these three lectins presenting higher affinities for α/β-N-acetylgalactosamine, α-mannose and N-acetylglucosamine, respectively (Elloway et al., 2004). These carbohydrate-containing vesicles were however probably distinct from the electron dense vesicles, mainly composed of lipids, observed at the periphery of cellulase-treated cysts in electron microscopy. These peripheral lipidic vesicles were significantly smaller than the ones observed in the whole cytoplasm of the untreated control. Conversely, the volume of lipid vesicles, presumably storing energy for the amoeba, has been shown to increase during *A. castellanii* encystment (Bowers and Korn, 1969). Therefore, during the process of excystment promoted by cellulase treatment, lipid vesicles would localize at the periphery of the amoeba and their content would be partially discharged to be potentially used as a source of energy by the excysting trophozoite.

In conclusion, in the present work, we have determined optimal conditions of cellulase, collagenase and pepsin treatment to promote *Acanthamoeba castellanii* excystment, in terms of temperature, time of contact and enzyme concentration. To the best of our knowledge, this work has never been performed before. While these conditions could not be transposed to other FLA, an additional effect was observed by treating *A. castellanii* cysts with these enzymes associated two by two. Following excystment, trophozoite growth was also markedly stimulated in the presence of cellulase, presumably due to the digestion of the main carbohydrate component, namely cellulose, of *Acanthamoeba sp.* cyst wall. At the ultrastructural level, excystment stimulation by cellulase treatment was characterized by endocyst disarrangement and intercystic space area reduction, allowing the trophozoite to come out of the cyst. In future works, other treatment conditions have to be determined to stimulate excystment of other FLA. A more detailed study of these cyst differentiation pathways would then be necessary in order to identify therapeutic targets to further develop new anti-amoebal drugs.

## Data Availability

The raw data supporting the conclusions of this article will be made available by the authors, without undue reservation.
