# Supplementary material for: Stimulation of Acanthamoeba castellanii excystment by enzyme treatment and consequences on trophozoite growth
Source: Front Cell Dev Biol. 2022 Sep 12;10:982897. doi: 10.3389/fcell.2022.982897 (PMC9511172; doi:10.3389/fcell.2022.982897)
Supplement: Supplementary file 1 [file Table1.DOCX]

**Supplementary Figure S1:** Effect of chitinase on *Acanthamoeba castellanii* excystment. Proportions of *A. castellanii* trophozoites and cysts observed following 24 h (A), 48 h (B) and 72 h (C) of chitinase treatment at 27°C as a function of enzyme concentration. The results correspond to the mean of four independent experiments ± SD.

**Supplementary Figure S2:** Effect of pepsin at pH 2 on *Acanthamoeba castellanii* excystment. Proportions of *A. castellanii* trophozoites and cysts observed following 1 day (A), 2 days (B) 3 days (C), 4 days (D), 11 days (E) and 18 days (F) of pepsin treatment at 30°C at 100 U/mL. Pepsin was used in regular PYG medium either at pH 6.5, or at pH 2. Total excystment in PYG medium alone at pH 6.5 and at pH 2, was observed at 11 days (E) and at 18 days (F), respectively. The results correspond to the mean of four replicates ± SD.


**Supplementary Figure S3:** Effect of cellulase treatment on *A. castellanii* endocyst labeling by FITC-lectins. Percentage of endocyst-labeled cysts following their treatment in the absence (black) or in the presence (grey) of 7.5 U/mL cellulase at 27°C for 48 h. Nine different lectins were used in this experiment: Con A, GSL II, Jacalin, LCA, LEL, SBA, SWGA, VVL and WGA. The results correspond to the mean of at least 25 cells ± SEM. Four independent experiments were performed.

**Supplementary Figure S4:** Labeling of SWGA lectin on *A. castellanii* trophozoites following their excystment. Trophozoites were labeled with FITC-SWGA following their excystment in the presence of 7.5 U/mL cellulase at 27°C for 48 h. Phase contrast and merge images are shown on the left and on the right the FITC-SWGA image, respectively. Scale bars = 10 µm.




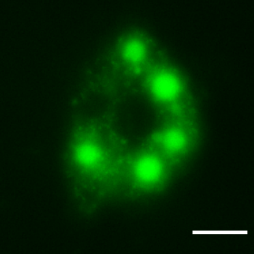

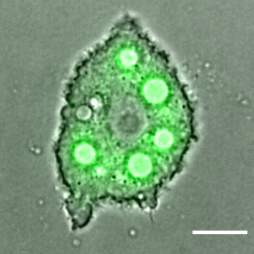


**Contrast**

**Lectin**

**Merge**
